# A novel prognostic model of de novo metastatic hormone-sensitive prostate cancer to optimize treatment intensity

**DOI:** 10.1007/s10147-024-02577-1

**Published:** 2024-07-19

**Authors:** Hiroshi Fujiwara, Masashi Kubota, Yu Hidaka, Kaoru Ito, Takashi Kawahara, Ryoma Kurahashi, Yuto Hattori, Yusuke Shiraishi, Yusuke Hama, Noriyuki Makita, Yu Tashiro, Shotaro Hatano, Ryosuke Ikeuchi, Masakazu Nakashima, Noriaki Utsunomiya, Yasushi Takashima, Shinya Somiya, Kanji Nagahama, Takeru Fujimoto, Kosuke Shimizu, Kazuto Imai, Takehiro Takahashi, Takayuki Sumiyoshi, Takayuki Goto, Satoshi Morita, Takashi Kobayashi, Shusuke Akamatsu

**Affiliations:** 1 Shizuoka City Shizuoka Hospital, Shizuoka, Japan; 2 Department of Urology, Kyoto University Graduate School of Medicine, Kyoto, Japan; 3 Department of Biomedical Statistics and Bioinformatics, Kyoto University Graduate School of Medicine, Kyoto, Japan; 4 Department of Urology, Miyazaki University Graduate School of Medicine, Miyazaki, Japan; 5 Department of Urology, Tsukuba University Graduate School of Medicine, Tsukuba, Japan; 6 Department of Urology, Faculty of Life Sciences, Kumamoto University, Kumamoto, Japan; 7 Department of Urology, Kobe City Medical Center General Hospital, Kobe, Japan; 8 Department of Urology, Shizuoka General Hospital, Shizuoka, Japan; 9 Department of Urology, Kurashiki Central Hospital, Kurashiki, Japan; 10 Department of Urology, Kyoto City Hospital, Kyoto, Japan; 11 Department of Urology, Red Cross Otsu Hospital, Otsu, Japan; 12 Department of Urology, Shimada General Medical Center, Shimada, Japan; 13 Department of Urology, Medical Research Institute Kitano Hospital, Osaka, Japan; 14 Department of Urology, Japanese Red Cross Wakayama Medical Center, Wakayama, Japan; 15 Department of Urology, Kobe City Nishi-Kobe Medical Center, Kobe, Japan; 16 Department of Urology, Tenri Hospital, Tenri, Japan; 17 Department of Urology, Takeda General Hospital, Kyoto, Japan; 18 Department of Urology, Rakuwakai Otowa Hospital, Kyoto, Japan; 19 Department of Urology, National Hospital Organization Himeji Medical Center, Himeji, Japan; 20 Department of Urology, Hamamatsu Rosai Hospital, Hamamatsu, Japan; 21 Department of Urology, Kansai Electric Power Hospital, Osaka, Japan; 22 Department of Urology, Osaka Red Cross Hospital, Osaka, Japan; 23 Department of Urology, Nagoya University Graduate School of Medicine, Nagoya, Japan

**Keywords:** Cancer-specific survival, mHSPC, Prognostic model, Prostate cancer

## Abstract

**Background:**

The treatment and prognosis of de novo metastatic hormone-sensitive prostate cancer (mHSPC) vary. We established and validated a novel prognostic model for predicting cancer-specific survival (CSS) in patients with mHSPC using retrospective data from a contemporary cohort.

**Methods:**

1092 Japanese patients diagnosed with de novo mHSPC between 2014 and 2020 were registered. The patients treated with androgen deprivation therapy and first-generation anti-androgens (ADT/CAB) were assigned to the Discovery (*N* = 467) or Validation (*N* = 328) cohorts. Those treated with ADT and androgen-receptor signaling inhibitors (ARSIs) were assigned to the ARSI cohort (*N* = 81).

**Results:**

Using the Discovery cohort, independent prognostic factors of CSS, the extent of disease score ≥ 2 or the presence of liver metastasis; lactate dehydrogenase levels > 250U/L; a primary Gleason pattern of 5, and serum albumin levels ≤ 3.7 g/dl, were identified. The prognostic model incorporating these factors showed high predictability and reproducibility in the Validation cohort. The 5-year CSS of the low-risk group was 86% and that of the high-risk group was 22%. Approximately 26.4%, 62.7%, and 10.9% of the patients in the Validation cohort defined as high-risk by the LATITUDE criteria were further grouped into high-, intermediate-, and low-risk groups by the new model with significant differences in CSS. In the ARSIs cohort, high-risk group had a significantly shorter time to castration resistance than the intermediate-risk group.

**Conclusions:**

The novel model based on prognostic factors can predict patient outcomes with high accuracy and reproducibility. The model may be used to optimize the treatment intensity of de novo mHSPC.

**Supplementary Information:**

The online version contains supplementary material available at 10.1007/s10147-024-02577-1.

## Introduction

Potent androgen receptor signaling inhibitors (ARSIs), along with androgen deprivation therapy (ADT), have become the standard treatment for metastatic hormone-sensitive prostate cancer (mHSPC), irrespective of tumor volume or risk. Several new classes of drugs, such as poly ADP-ribose polymerase inhibitors, AKT inhibitors, and 177 Lu-PSMA, are currently being tested in clinical trials to examine whether the addition of these agents to the current standard of care (SOC), “ARSI and ADT doublet”, can improve the survival of patients with mHSPC [1, 2]. Recently, two clinical trials, ARASENS and PEACE-1, showed that the addition of darolutamide or abiraterone to another SOC, docetaxel and ADT, improved the overall survival (OS) of patients with mHSPC, regardless of tumor volume or risk [3, 4]. However, considering the side effects of taxane chemotherapy, it is debatable whether these triplet therapies should be considered over ARSI and ADT doublet in all patients with mHSPC.

Another recent trend in mHSPC management is a multi-modal treatment that incorporates radiation therapy. Large clinical trials have shown that radiation to the primary prostate cancer in addition to systemic therapy improves the OS of patients with oligometastatic mHSPC [5, 6]. Although robust evidence does not exist, several clinical trials are ongoing to test whether radiation to the metastatic sites (metastasis-directed therapy [MDT]) improves survival in oligometastatic mHSPC [7, 8]. In the future, it may become possible to de-escalate systemic therapy (stopping ARSI or even ADT after certain periods) by combining it with radiation to the primary tumor and all metastatic sites in some patients [9]. However, appropriate risk assessment is warranted to optimally escalate or de-escalate treatment.

The CHAARTED tumor volume and LATITUDE risk classifications are the most widely used risk classifications for mHSPC [10, 11]. However, unlike the International Metastatic Renal-Cell Carcinoma Database Consortium (IMDC) risk score for renal cell carcinoma, these risk classifications were arbitrarily constructed and were not based on multivariable analyses incorporating many potential prognostic factors. We have previously conducted a retrospective analysis of 304 Japanese patients with de-novo mHSPC and identified that among major clinical parameters, the extent of disease score (EOD) ≥ 2 or the presence of liver metastasis; lactate dehydrogenase levels > 250U/L; and a primary Gleason pattern of 5 were independently associated with prognosis [12]. We established a risk classification model (Kyoto model) based on these three factors and validated it using a cohort of 520 patients. The Kyoto model was able to reclassify both CHAARTED high- and low-volume patients into three risk groups with significantly different OS, showing its superiority over the existing criteria (similar data were obtained in comparison with the LATITUDE risk classification; however, the data have not been published). The major limitation of the study was that the discovery cohort consisted of a heterogeneous population, including patients who were treated before the availability of ARSIs and taxanes, and the reproducibility of the model was moderate (Harrell’s C-index, 0.649). For a more precise risk classification that can be used to escalate or de-escalate treatment in the present era, in the present study, we updated the Kyoto model using the data of an independent cohort of patients who were diagnosed after 2014, when both abiraterone and enzalutamide as well as cabazitaxel became available in Japan.

## Material and methods

### Patients

This was a multicenter, retrospective observational study of patients with de novo mHSPC (synchronous mHSPC) who were diagnosed and started treatment between 2014 and 2020. Clinical data were collected on 620 patients from 15 sites in January 2022 to form Cohort 1. After creating a prognostic model for mHSPC in cohort 1, additional clinical data were collected from 7 other centers to validate the risk model, making it Cohort 2. Cohort 2 enrolled 472 cases in December 2022. This study was approved by the institutional ethics committees of each institution, and written informed consent was waivered because of the retrospective design. All procedures involving human participants were conducted in accordance with the ethical standards of the Institutional Research Committee and the 1964 Helsinki Declaration and its later amendments or comparable ethical standards.

All patients had distant metastases identified using computed tomography or bone scans at diagnosis. From cohorts 1 and 2, we excluded 107 and 109 patients, respectively, with (1) less than 3 months of follow-up period; (2) unknown/undetermined Gleason score, EOD scores, or laboratory data at initial diagnosis; (3) docetaxel use at initial treatment, which was not covered by public insurance in Japan during the study period (*N* = 4 and 7 in cohorts 1 and 2, respectively); or (4) non-available prognosis data.

### Clinical data acquisition and definition of survival endpoints

Demographic, clinicopathological, and survival data were obtained from the medical records. EOD was used as a semiquantitative grading system according to the extent of bone metastasis on bone scans as follows: 0, normal; 1, fewer than six bony metastases, each of which is < 50% of the size of a vertebral body; 2, between six and 20 bony metastases; 3, more than 20 bony metastases but less than a “super scan”; and 4, “super scan” or bony metastases involving more than 75% of the ribs, vertebrae, and pelvic bones [13]. Cancer-specific survival (CSS) was defined as the time from treatment initiation to death due to prostate cancer (PCa). Castration resistance was defined as prostate specific antigen (PSA) progression based on the Prostate Cancer Clinical Trial Working Group 2 and/or radiological and/or clinical progression despite a serum total testosterone level < 50 ng/dL. The time to castration-resistant prostate cancer (CRPC) was defined as the time from treatment initiation until diagnosis of castration resistance. Patients who survived were censored at their last follow-up visit. The cases were also risk-classified according to the criteria in the CHARRTED and the LATITUDE studies.

### Study design

**Fig. 1:**
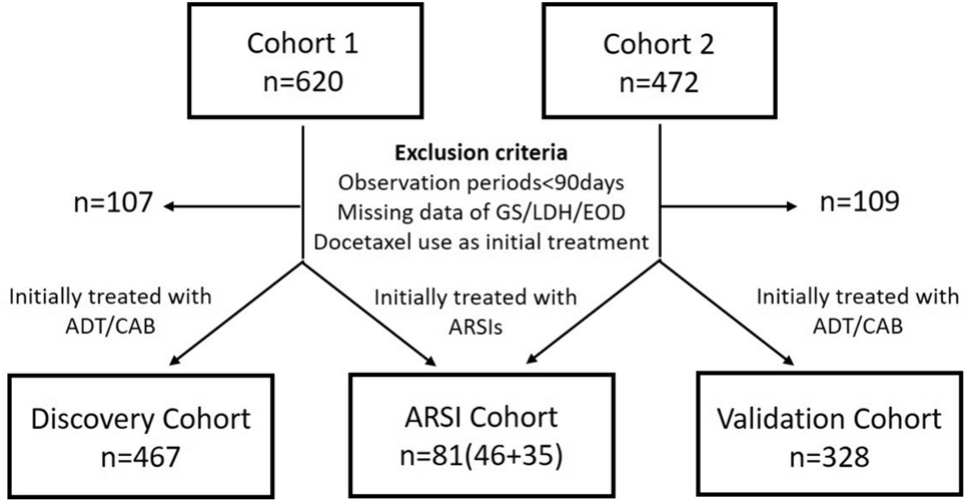
Flowchart of the study enrollment

Since the number of patients initially treated with ARSI was still small during this study period, we first focused on patients treated with ADT alone or combined androgen deprivation therapy (CAB) with bicalutamide or flutamide. The patients were assigned to the Discovery and Validation cohorts, excluding those initially treated with ARSI from Cohorts 1 and 2. The patients initially treated with ARSI in Cohort 1 and 2 were combined and assigned to the ARSI cohort (Fig. 1). The Discovery, Validation, and ARSI cohorts comprised 467, 328, and 81 patients, respectively. First, a new prognostic model was developed for mHSPC patients initially treated with ADT alone or CAB using the Discovery cohort, and then the model was validated using the Validation cohort. Finally, we tested whether this risk model could also stratify the prognosis of patients who received initial ARSI treatment using ARSI cohort.

### Statistical analysis

Statistical analyses were performed using EZR (Saitama Medical Center, Jichi Medical University, Saitama, Japan), a graphical user interface for R. More precisely, it is a modified version of the R commander designed to add statistical functions frequently used in biostatistics [14]. Continuous variables are reported as means ± standard deviations or medians and interquartile ranges (IQRs). Categorical variables are reported as numbers and percentages. Survival analyses were conducted using the Kaplan–Meier method and the log-rank test. Univariate Cox proportional hazards models were used to examine the associations between CSS and potential prognostic factors, with variables selected for multivariable analysis using the forward stepwise selection method (*p* < 0.05). Linear predictors extracted from the Cox models were used to develop risk scores, and risk categories were defined based on the hazard ratios and the frequency of each risk score. Discrimination was evaluated using Harrell’s C-index [15]. The predictive ability of the novel model and the original Kyoto model for CSS was evaluated using continuous net reclassification improvement (NRI) and integrated discrimination improvement (IDI) [16]. Validation was performed by applying data from the Validation and ARSI cohorts to the regression coefficients obtained during model development.

## Results

### Patient characteristics

Table 1 presents the characteristics of the patients included in the Discovery, Validation, and ARSI cohorts. The patients’ backgrounds in the Discovery and Validation cohorts were generally similar. Over 90% of the patients had an International Society of Urological Pathology (ISUP) Grade Group of ≥ 4. and there were more patients with primary Gleason pattern of 5 in the Validation cohort than in the Discovery cohort (21.2% vs. 26.5%, *p* = 0.089). There were more patients with bone metastases of EOD ≥ 2 in the Validation cohort (47.1% vs. 55.5%, *p* = 0.021). Patients in the ARSI cohort showed higher initial PSA levels and EOD scores, and there were more patients with primary Gleason pattern of 5 and visceral metastasis compared to the Discovery and Validation cohorts. In Japan, abiraterone for high-risk mHSPC was covered by public insurance in 2016. Therefore, the most common ARSI used was abiraterone (90.1%).

**Table 1:** Characteristics of the study population

	Discovery cohort	Validation cohort	ARSI cohort
	*n* = 467	*n* = 328	*n* = 81
Median age at diagnosis, years (range)	74 (45–90)	75 (52–97)	71 (40–90)
ECOG performance status, *n* (%)			
0–1	414 (88.7)	289 (88.1)	68 (84.0)
2–4	52 (11.2)	33 (11.0)	4 (4.9)
Median initial PSA, ng/ml (IQR)	224 (47.3–755)	250 (76.8–797)	473 (141–1588)
ISUP Grade group, *n* (%)			
1–3	41 (8.8)	20 (6.1)	2 (2.5)
4	197 (42.2)	137 (41.8)	22 (27.2)
5(4 + 5)	132 (28.3)	86 (26.2)	25 (30.9)
5(5 + 4)	59 (12.6)	47 (14.3)	15 (18.5)
5(5 + 5)	38 (8.1)	38 (11.6)	17 (21.0)
Pain at diagnosis, *n* (%)	99 (21.2)	69 (22.9)	23 (28.4)
Metastatic site, *n* (%)			
Regional lymph node	285 (61.0)	184 (56.1)	58 (71.6)
Distant lymph node	167 (35.8)	89 (27.1)	35 (43.2)
Bone	401 (85.9)	303 (92.4)	77 (95.1)
Lung	84 (18.0)	32 (10.6)	23 (28.4)
Liver	16 (3.4)	10 (3.3)	0 (0)
Extent of disease, *n* (%)			
0	66 (14.1)	25 (7.6)	4( 4.9)
1	181 (38.8)	121 (36.9)	12 (14.8)
2	97 (20.8)	92 (28.0)	17 (21.0)
3	101 (21.6)	71 (21.6)	35 (43.2)
4	22 (4.7)	19 (5.8)	13 (16.1)
Year of treatment start, *n* (%)			
2014	51 (10.9)	36 (11.0)	0 (0)
2015	74 (15.8)	58 (17.7)	0 (0)
2016	85 (18.2)	55 (16.8)	1 (2.2)
2017	104 (22.2)	66 (20.1)	5 (6.2)
2018	91 (19.4)	81 (24.7)	35 (43.2)
2019	55 (11.8)	29 (8.8)	29 (35.8)
2020	7 (1.5)	3 (0.9)	11 (13.6)
CHARRTED criteria, *n* (%)			
Low volume	202 (43.2)	129 (39.3)	13 (16.0)
High volume	255 (54.6)	199 (60.7)	68 (84.0)
unknown	10 (2.1)	0 (0)	0 (0)
LATITUDE criteria, *n* (%)			
Low risk	193 (41.3)	99 (30.2)	8 (9.9)
High risk	274 (58.7)	226 (68.9)	73 (90.1)
unknown	0 (0)	3 (0.9)	0 (0)
Median laboratory value at diagnosis (IQR)			
Hemoglobin (g/dl)	13.3 (12.0–14.6)	12.9 (11.6–14.2)	13.4 (12.3–14.3)
Lactate dehydrogenase (U/l)	199 (172–238)	202 (174–240)	214 (176–259)
Alkaline phosphatase (U/l)	315 (226–686)	362 (247–726)	536 (309–1168)
Albumin (g/dl)	4.0 (3.7–4.2)	4.0(3.6–4.2)	3.9 (3.5–4.2)
Initial treatment, *n* (%)			
Androgen deprivation therapy	70 (15.0)	63 (19.2)	–
Combined androgen blockade	397 (85.0)	265 (80.8)	–
Abiraterone	–	–	73 (90.1)
Enzalutamide	–	–	4 (4.9)
Apalutamide	–	–	4 (4.9)

*PSA* prostate specific antigen, *ISUP* International Society of Urological Pathology

### Creation of the CSS prognostic model

In the Discovery cohort, the median follow-up time was 37.8 months (IQR, 23.2–52.7), during which 126 (27.0%) patients died of PCa, and 318 (68.1%) were diagnosed with CRPC. Although the original Kyoto model was created to predict the OS, in the present study, we focused on the CSS to better represent the characteristics of PCa. In the Cox proportional hazards model analysis, the C-statistic for predicting the 5-year CSS was 0.690 when the original Kyoto model was applied to the Discovery cohort. In the Discovery cohort, univariate and multivariable analyses of various clinical parameters showed that in addition to the clinical variables used to create the original Kyoto model, serum albumin levels at diagnosis were significantly associated with the CSS (Table 2). The cutoff for albumin level was determined to be 3.7 g/dl using time-dependent receiver operating characteristics analysis (Figure S1). Because of the small number of patients with liver metastases at diagnosis (*n* = 16) was clearly smaller than the number of patients having other risks, we decided to group EOD ≥ 2 and liver metastases together in terms of metastatic volume. These two factors were shown to have similar hazards ratio for OS in our previous study establishing the original Kyoto model. Cox proportional hazards model analysis showed that an initial albumin level ≤ 3.7 g/dl was independently associated with the CSS, along with the other prognostic factors used to create the original Kyoto model (Table 3). The C-index for predicting the 5-year CSS was 0.73 for the Discovery cohort. Comparing the performance of the original Kyoto model with the novel model incorporating serum albumin levels in their ability to predict the 5-year CSS, the predictability of the novel model was significantly improved with a continuous NRI of 0.37 (IQR, 0.24–0.51; *p* < 0.0001) and IDI of 0.08 (IQR, 0.03–0.16; *p* < 0.0001) (Table 4).

**Table 2:** Univariable and multivariable Cox regression analyses for the prediction of CSS in the Discovery cohort

Parameter	Univariable		Multivariable	
	HR (95% CI)	*p*-value	HR (95% CI)	*p*-value
Age < 75	1.19 (0.83, 1.69)	0.346		
ECOG performance status > 1	2.36 (1.47, 3.78)	< 0.001		
Pain at diagnosis	2.25 (1.54, 3.29)	< 0.001		
PSA > 200 ng/ml	1.90 (1.32, 2.74)	< 0.001		
Primary Gleason pattern 5	2.38 (1.66, 3.42)	< 0.001	1.93 (1.30, 2.88)	0.001
EOD ≥ 2	2.82 (1.93, 4.12)	< 0.001	1.95 (1.25, 3.05)	0.003
Hemoglobin < 13.0 g/dl	1.70 (1.19, 2.41)	0.003		
Lactate dehydrogenase > 250U/l	3.37 (2.34, 4.85)	< 0.001	1.57 (1.01, 2.45)	0.047
Alkaline phosphatase > 300 U/l	2.52 (1.71, 3.70)	< 0.001		
Albumin ≤ 3.7 g/dl	3.95 (2.76, 5.66)	< 0.001	3.00 (2.02, 4.47)	< 0.001
Lung metastasis	0.82 (0.50, 1.34)	0.433		
Liver metastasis	3.45 (1.75, 6.82)	< 0.001	2.12 (1.00, 4.50)	0.049

*EOD* extent of disease, *GS* Gleason score, *LDH* lactate dehydrogenase, *ALB* albumin

**Table 3:** Multivariable Cox regression analyses for the prediction of CSS in the Discovery cohort

	Hazard Ratio	95% CI	*p*-value
ALB ≤ 3.7	2.81	1.92, 4.10	< 0.001
LDH > 250	1.75	1.17, 2.61	0.007
EOD ≥ 2 or Liver mets	1.82	1.19, 2.80	0.006
Primary Gleason pattern 5	1.94	1.34, 2.82	< 0.001

*EOD* extent of disease, *GS* Gleason score, *LDH* lactate dehydrogenase, *ALB* albumin

**Table 4:** Evaluation of the predictive ability of CSS in the original Kyoto model and the Modified Kyoto model

	Model	C-Statistics (95% CI)	Continuous NRI (95% CI)	p-value	IDI(95% CI)	p-value
1Year Risk	Kyoto model	0.78 (0.67, 0.94)				
	Modified Kyoto model	0.85 (0.81, 0.99)	0.53 (0.31, 0.69)	< 0.0001	0.02 (0.00, 0.04)	0.0199
3Year Risk	Kyoto model	0.70 (0.64, 0.75)				
	Modified Kyoto model	0.73 (0.66, 0.80)	0.34 (0.22, 0.48)	0.0100	0.05 (0.01, 0.10)	0.0100
5Year Risk	Kyoto model	0.69 (0.65, 0.72)				
	Modified Kyoto model	0.73 (0.66, 0.77)	0.37 (0.24, 0.51)	< 0.0001	0.08 (0.03, 0.16)	< 0.0001

*NRI* Net Reclassification Index, *IDI* Integrated Discrimination improvement Index

**Fig. 2:**
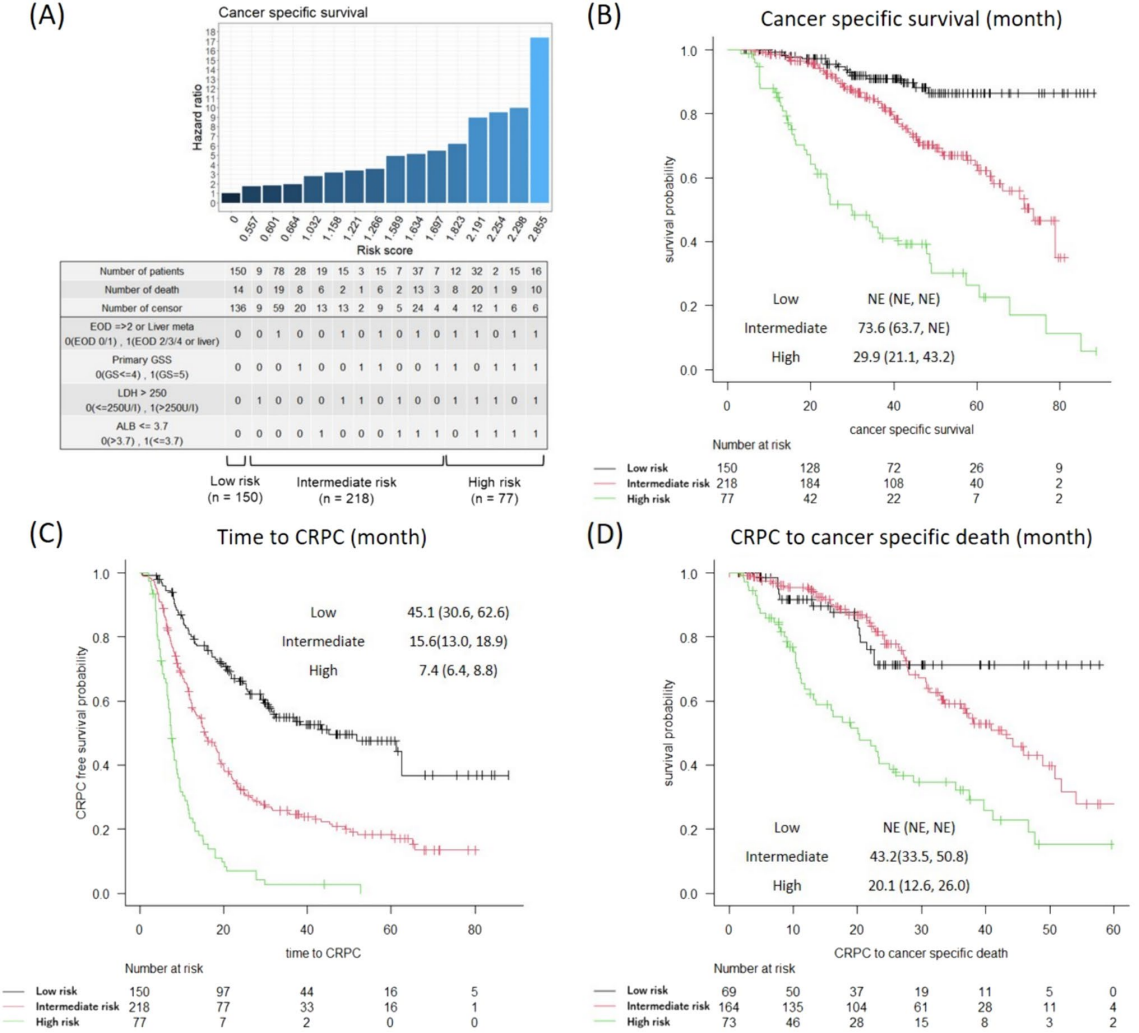
**A** Risk stratification based on the distribution of the predicted hazard ratios. Each combination of risk factors (x-axis) was scored according to the regression coefficients from the multivariable Cox regression model (Table 2). The y-axis shows the hazard ratio calculated for each combination. Kaplan–Meier curves for the CSS (**B**), time to CRPC (**C**), and CSS after castration resistance (**D**) according to the risk stratification

Next, we assigned one point to each of the four risk factors and calculated the hazard ratio for each risk score (Fig. 2A). The patients were classified into three risk groups according to the total score: 0 points for the low-risk group, 1–2 points for the intermediate-risk group, and 3–4 points for the high-risk group (Modified Kyoto model). According to the model, the number of patients in the high-, intermediate-, and low-risk groups was 77 (17.3%), 218 (49.0%), and 150 (33.7%), respectively. There was a significant difference in the CSS between the risk groups (Fig. 2B). The median CSS for the high- and intermediate-risk groups was 29.9 months (IQR, 21.1–43.2) and 73.6 months (IQR, 63.7–Not Evaluable [NE]), respectively, and was not reached for the low-risk group (*p* < 0.001). For each risk group, we also examined the differences in the time to CRPC as well the CSS after becoming castration-resistant. The median times to CRPC in the high-, intermediate-, and low-risk groups were 7.4, 16, and 45 months, respectively (*p* < 0.001, Fig. 2C). The high-risk group not only had a shorter time to CRPC but also had a significantly worse CSS after castration resistance (median, 20.1 months; IQR, 12.6–26.0) than the non-high-risk groups (*p* < 0.001, Fig. 2D).

### Validation of the novel prognostic model

In the Validation cohort, the median follow-up time was 41.3 months (IQR, 25.1–59.3), during which 77 (23.5%) patients died of prostate cancer, and 194 (59.1%) were diagnosed with CRPC. The Cox proportional hazards model analysis showed that an initial albumin level ≤ 3.7 g/dl was also significantly associated with the CSS (Table 5). Moreover, the risk model incorporating the albumin level showed high reproducibility in the Validation cohort; the C-statistics for predicting the 5-year CSS was 0.76.

**Table 5:** Multivariable Cox regression analyses for the prediction of CSS in the Validation cohort

	Hazard Ratio	95% CI	*p*-value
ALB ≤ 3.7	2.11	1.31, 3.41	0.002
LDH > 250	2.39	1.48, 3.85	< 0.001
EOD ≥ 2 or Liver mets	3.02	1.70, 5.36	< 0.001
Primary Gleason pattern 5	1.68	1.03, 2.72	0.037

*EOD* extent of disease, *GS* Gleason score, *LDH* lactate dehydrogenase, *ALB* albumin

**Fig. 3:**
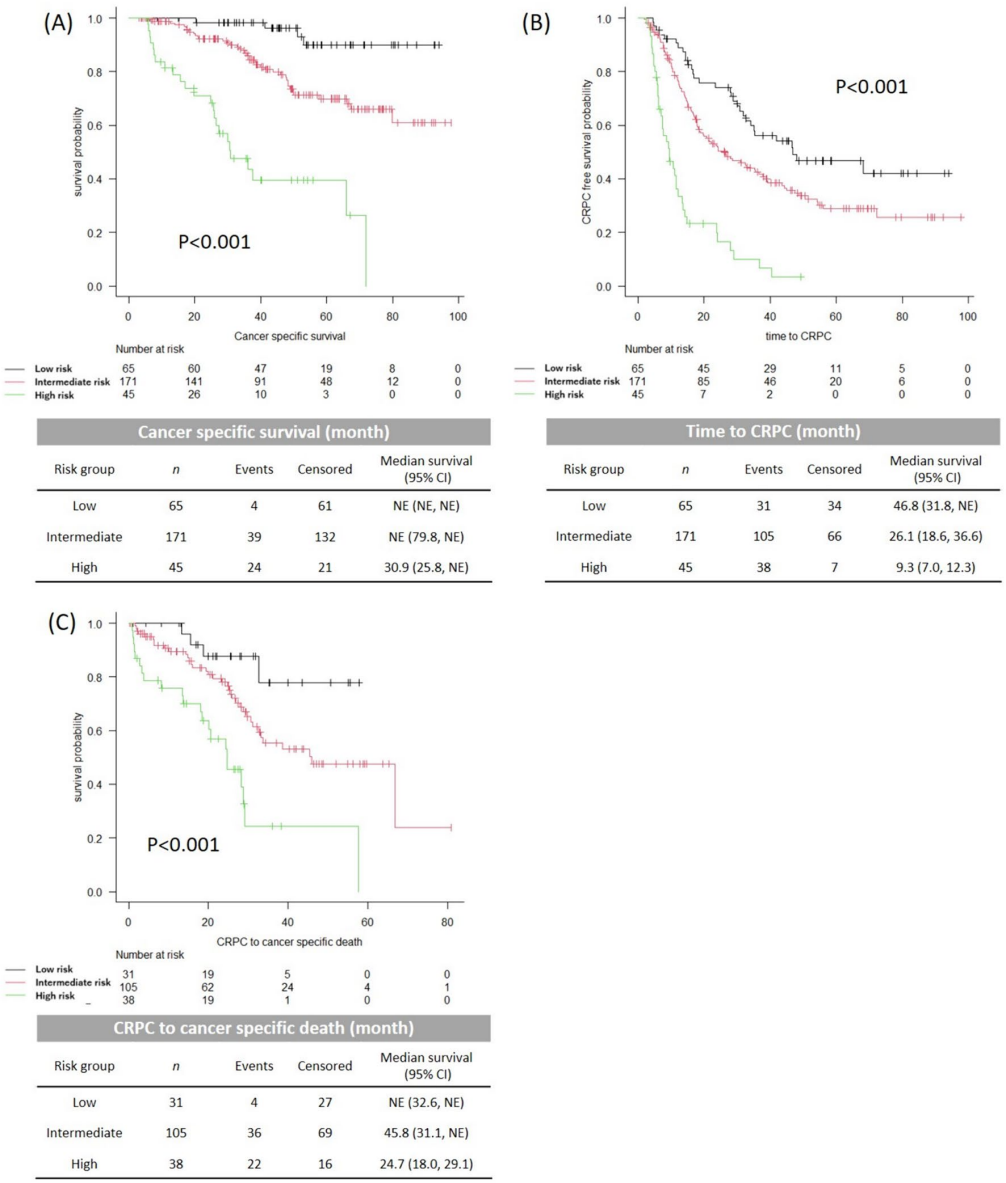
Kaplan–Meier curves for CSS (**A**), Time to CRPC (**B**) and CSS after castration resistance (**C**) according to the risk stratification in the Validation cohort

According to the Modified Kyoto model, the number of patients in the high-, intermediate-, and low-risk groups was 45 (14.5%), 171 (55.0%), and 95 (30.5%), respectively. The median CSS for the high-risk group was 30.9 months (IQR, 25.8–NE) and was not reached for the intermediate- and low-risk groups (*p* < 0.001, Fig. 3A). Similar to the Discovery cohort, there were significant differences in the time to CRPC and the CSS after castration resistance between the risk groups (Fig. 3B, C).

### Reclassification of the LATITUDE risk groups by the novel prognostic model

**Fig. 4:**
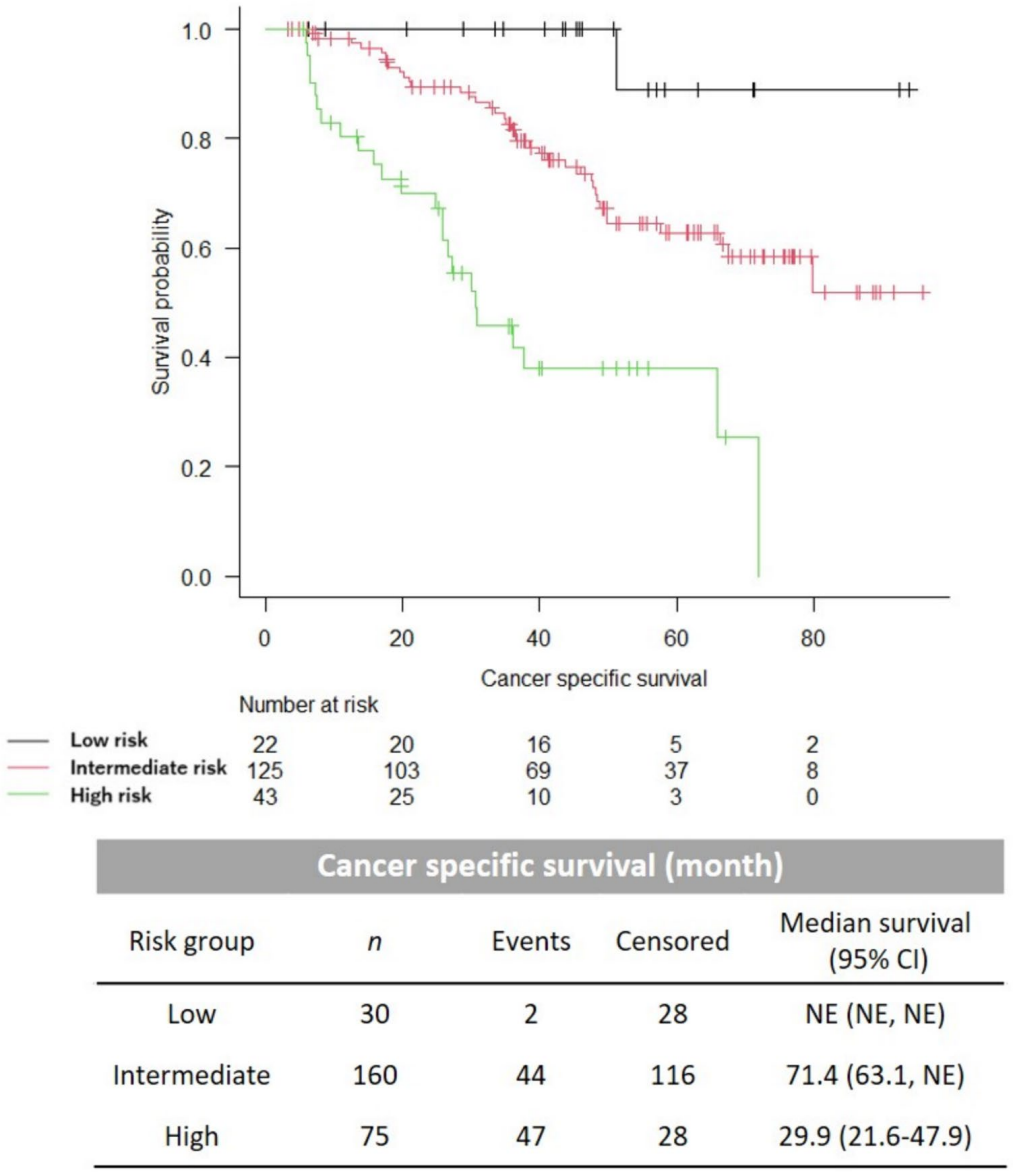
Kaplan–Meier curves for the CSS after reclassification of the LATITUDE high-risk patients by the Modified Kyoto model in the Validation cohort

Next, we examined whether the Modified Kyoto model could further stratify the LATITUDE high- and low-risk groups. Approximately 22.6, 65.8, and 11.6% of the patients in the Validation cohort defined as high-risk by the LATITUDE criteria were grouped into high-, intermediate-, and low-risk groups by the Modified Kyoto model, respectively (Table 6). The prognosis of each group was also clearly stratified, and median CSS was 29.9 and 71.4 months in the high- and intermediate-risk groups, respectively, and was not reached in the low-risk group (*p* < 0.001, Fig. 4).

**Table 6:** Stratification of LATITUDE high and low risk groups by the Modified Kyoto model

	Modified Kyoto model		
	Low	Intermediate	High
LATITUDE-Low	42 (47.7)	44 (50.0)	2 (2.3)
LATITUDE-High	22 (11.6)	125 (65.8)	43 (22.6)

### Validation in the ARSI cohort

**Fig. 5:**
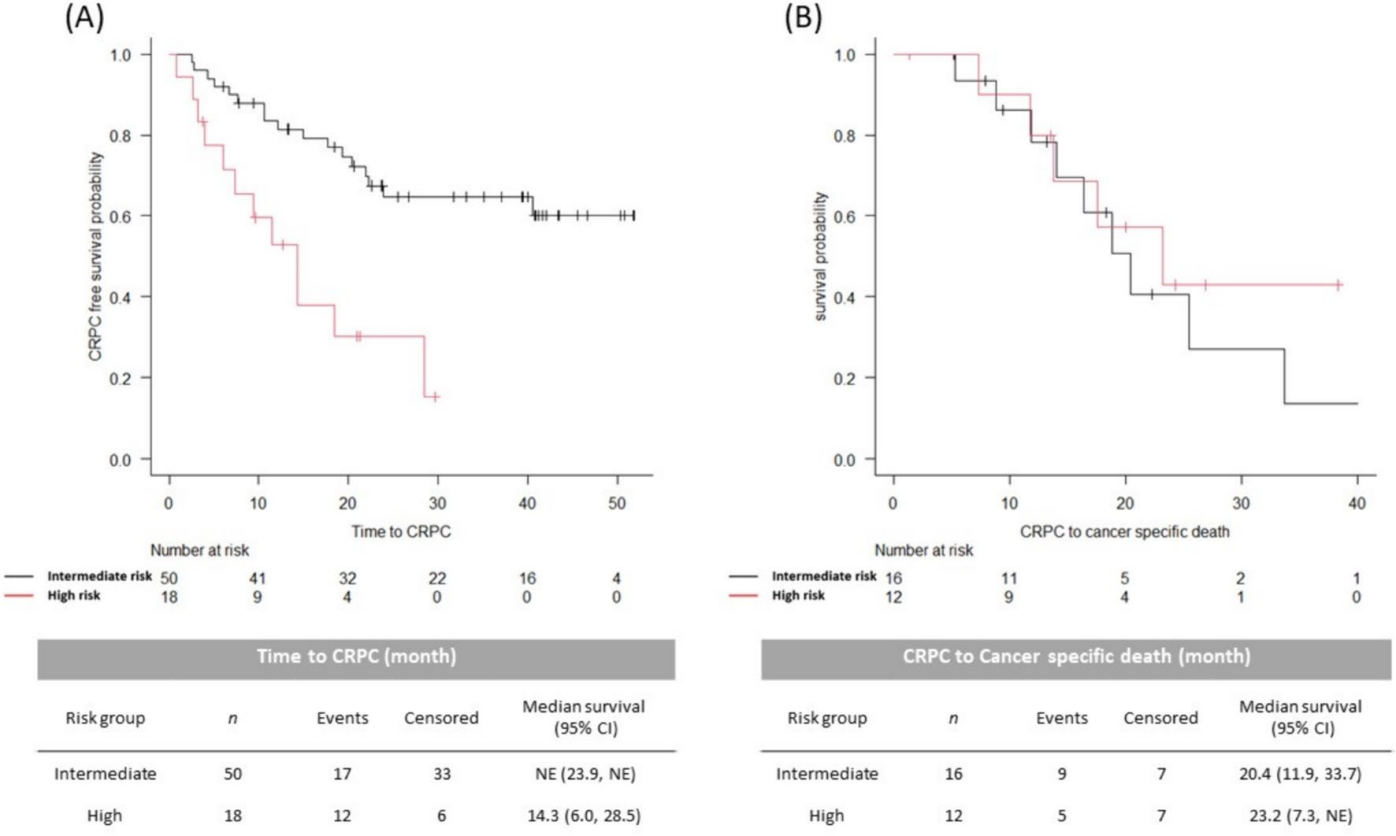
Kaplan–Meier curves for the time to CRPC (**A**) and CSS after castration resistance (**B**) of the intermediate- and high-risk patients in the ARSI cohort

In the ARSI cohort, the median follow-up time was 31.8 months (IQR, 17.3–40.6). According to the Modified Kyoto model, the number of patients in the high-, intermediate-, and low-risk groups was 18, 50, and 7, respectively. During the follow-up time, 5(62.5%), 11(22.0%) and 0 patients died of prostate cancer, and 12(66.7%), 16(32.0%) and 1(14.3%) were diagnosed with CRPC. High-risk group had a significantly shorter time to CRPC than the intermediate-risk group (NE vs. 14.3, *p* < 0.001), however, CSS after castration resistance was similar (20.4 vs. 23.2, *p* = 0.577). (Fig. 5) Median CSS was not reached in either group during the follow-up.

## Discussion

The prognosis of patients with mHSPC varies. While some patients develop CRPC within a year of ARSI and ADT doublet therapy, others show a sustained response to ADT/CAB for more than 5 years, as shown in the present study. Triplet therapy combining ARSI and taxane is one way to intensify therapy. In the present study, the high-risk group in the Modified Kyoto model not only progressed to CRPC earlier but also had shorter survival after castration resistance. It has recently been reported that prostate cancer with certain genomic alterations such as *BRCA2*, *CDK12*, *TP53*, and *RB1* have very poor prognosis [17, 18]. These patients not only become castration resistant earlier, but also have poor outcome after becoming CRPC. Since approximately 15% of patients were classified as high-risk by the Modified Kyoto model both in the discovery and validation cohorts, these patients are likely to be enriched with genomic alterations associated with poor outcomes, and may benefit from more intensified initial therapy combining ARSI with chemotherapy or other drugs such as PARP inhibitors. On the other hand, in the low-risk group, the median time to castration resistance was 45.1 months, and the 5-year CSS approached 90%, even with initial treatment with ADT/CAB. It is expected that many of these patients, when initially treated with the ARSI and ADT doublet, will remain on the drugs for more than 5 years. Considering the financial burden and long-term side effects of ARSI and ADT, such as cognitive function impairment and fractures, it is reasonable to consider the de-escalation of systemic therapy, especially in oligometastatic patients whose disease sites have been adequately controlled by local treatment, including MDT.

In the present study, we constructed a highly reproducible prognostic model consisting of metastatic volume and site (EOD ≥ 2 or presence of liver metastasis), pathological features (primary Gleason pattern of 5), and serum markers (LDH and albumin). Notably, these parameters were chosen from the results of a multivariable analysis in a manner similar to the IMDC risk score in renal cell carcinoma. Using this model, we showed that the LATITUDE high- and low-risk groups can be further divided into three risk groups with significantly different CSS. Currently, subgroup analyses in many clinical trials have shown that a target drug has a similar effect in both LATITUDE high- and low-risk groups or CHAARTED high- and low-volume disease [19–22]. However, our data clearly show that these existing risk groups comprise heterogeneous populations with different prognoses. Thus, showing similar effects in these subgroups in a clinical trial is not sufficient to demonstrate that the drug has similar clinical importance in all patients. For optimal patient selection in a real-world setting, a more precise risk classification, such as the Modified Kyoto model, is necessary. There are several differences between the Modified Kyoto model and the LATITUDE risk classification. First, in the LATITUDE risk classification, all visceral metastases were considered poor prognostic factors; however, in the Modified Kyoto model, only liver metastases were considered poor prognostic factors among visceral metastases. Second, regarding the pathological grade, we focused on the primary Gleason pattern. In the original Kyoto model, as well as in the Modified Kyoto model, we have consistently shown that a prognostic difference exists between Gleason scores of 4 + 4 or 4 + 5 and 5 + 4 or 5 + 5 and that a primary Gleason pattern of 5 is an independent prognostic factor (Figure S2). Third, serum markers were incorporated as independent prognostic factors. The albumin level had a strong impact on the OS in our previous study, which reported the original Kyoto model; however, it was not included in the prognostic model because of the lack of albumin data in the validation cohort.

This study had several limitations. First, this was a retrospective study, and clinical, pathological, and radiographic evaluations were performed by specialists at each institution. Second, in Japan, the current SOC for mHSPC is ARSI doublet and triplet therapy, but the prognostic model was based on data from patients who received ADT/CAB. Recently, an interim analysis of a large registry trial of high-risk mHSPC, J-ROCK, was reported, which showed that ARSIs prolong PFS and OS in Japanese patients [23]. Currently, we are conducting a prospective study on mHSPC diagnosed after 2020, which is expected to include more patients treated with ADT and ARSI doublet. The performance of the Modified Kyoto model will be further evaluated using this cohort. Lastly, next-generation imaging modalities, such as PSMA-PET and whole-body MRI, were not used as diagnostic tools. PSMA-PET is still not available in Japan, which may have led to an underestimation of the volume of metastasis. When PSMA-PET becomes the standard in Japan, it will be necessary to re-evaluate the prognostic model.

## Conclusions

In the contemporary cohort receiving ADT/CAB as the initial treatment for mHSPC, the independent prognostic factors of mHSPC (EOD ≥ 2 or the presence of liver metastasis; a primary Gleason pattern of 5; and serum LDH and albumin levels) were consistent among Japanese, and the predictability of prognosis by the Modified Kyoto model was high. The model was also able to predict the duration of response in the cohort initially treated with ARSI. The Modified Kyoto model may help guide the intensification of treatment in patients with mHSPC in the current era.

## Supplementary Information

Below is the link to the electronic supplementary material.

Supplementary file1 (DOCX 169 KB)
