# Supplementary material for: A novel prognostic model of de novo metastatic hormone-sensitive prostate cancer to optimize treatment intensity
Source: Int J Clin Oncol. 2024 Jul 19;29(10):1574–85. doi: 10.1007/s10147-024-02577-1 (PMC11420339; doi:10.1007/s10147-024-02577-1)

FigureS1. Time dependent ROC curve for albumin in predicting cancer specific survival


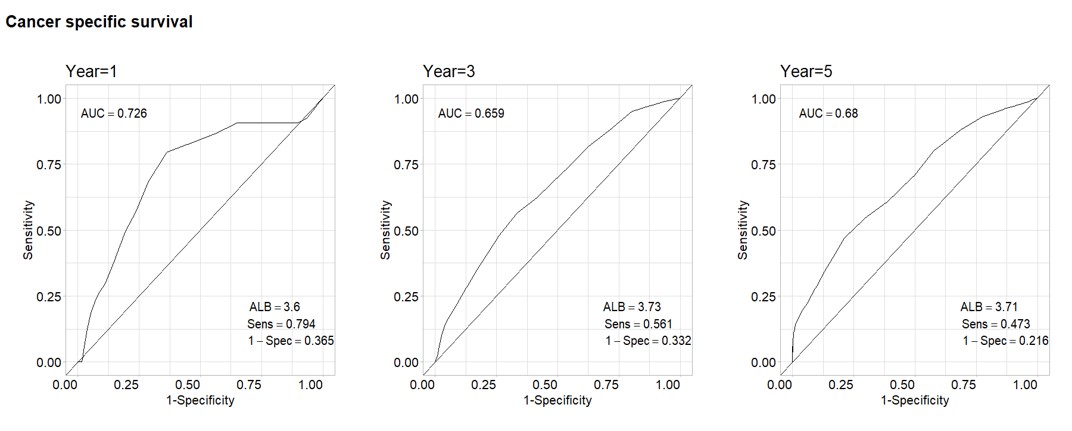


FigureS2. Kaplan-Meier curves for CSS according to the ISUP Grade Group.


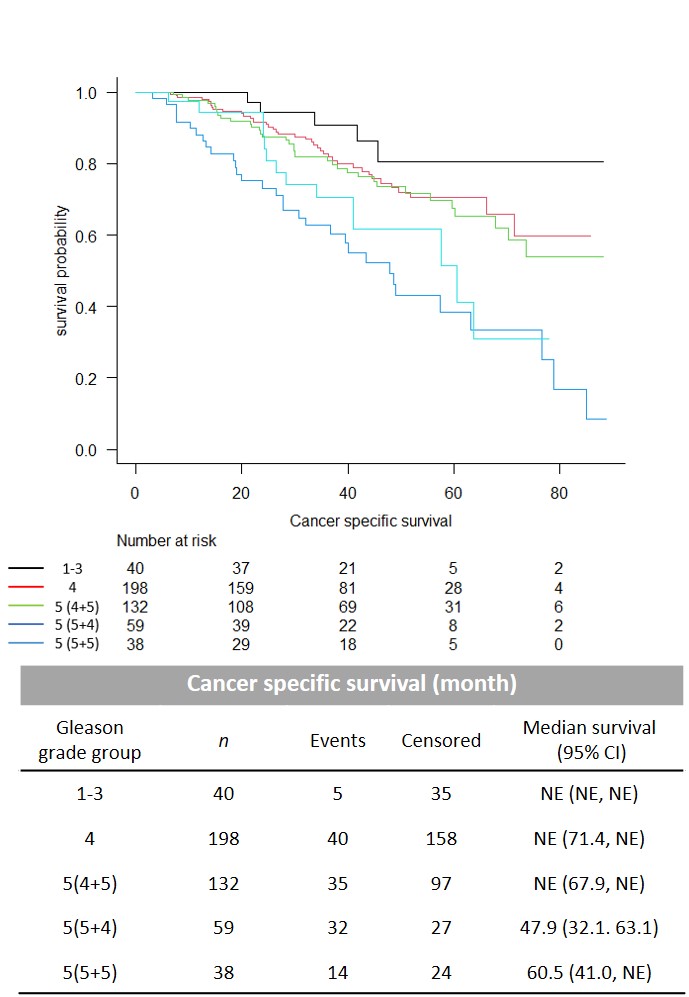

Supplement: Supplementary file 1 — Supplementary file1 (DOCX 169 KB) [file 10147_2024_2577_MOESM1_ESM.docx]
